# A phase II study of alternating sunitinib and temsirolimus therapy in patients with metastatic renal cell carcinoma

**DOI:** 10.1002/cam4.5990

**Published:** 2023-05-06

**Authors:** Dylan B. Ness, Darcy B. Pooler, Steven Ades, Brian J. Highhouse, Bridget M. Labrie, Jie Zhou, Jiang Gui, Lionel D. Lewis, Marc S. Ernstoff

**Affiliations:** ^1^ Department of Medicine and the Dartmouth Cancer Center at Dartmouth‐Hitchcock Medical Center Section of Clinical Pharmacology Lebanon New Hampshire USA; ^2^ Division of Hematology/Oncology University of Vermont Cancer Center Burlington Vermont USA; ^3^ Section of Hematology/Oncology and the Dartmouth Cancer Center at Dartmouth‐Hitchcock Medical Center Lebanon New Hampshire USA; ^4^ Department of Biomedical Data Science and the Geisel School of Medicine at Dartmouth Lebanon New Hampshire USA; ^5^ Developmental Therapeutics Program, Division of Cancer Treatment and Diagnosis at National Cancer Institute ImmunoOncology Branch Bethesda Maryland USA

**Keywords:** clinical cancer research, clinical trials, renal cancer, target therapy

## Abstract

**Background:**

Sunitinib is a multi‐target tyrosine kinase inhibitor (TKI) that inhibits VEGF receptor 1, 2, 3 (VEGFRs), platelet‐derived growth factor receptor (PDGFR), colony‐stimulating factor receptor (CSFR), and the stem cell factor receptor c‐KIT. Temsirolimus inhibits mammalian target of rapamycin (mTOR) through binding to intracellular protein FKBP‐12. Both agents are approved for the treatment of metastatic renal cell carcinoma (mRCC), have different anticancer mechanisms, and non‐overlapping toxicities. These attributes form the scientific rationale for sequential combination of these agents. The primary objective of the study was to investigate the efficacy of alternating sunitinib and temsirolimus therapy on progression‐free survival (PFS) in mRCC.

**Methods:**

We undertook a phase II, multi‐center, single cohort, open‐label study in patients with mRCC. Patients were treated with alternating dosing of 4 weeks of sunitinib 50 mg PO daily, followed by 2 weeks rest, then 4 weeks of temsirolimus 25 mg IV weekly, followed by 2 weeks rest (12 weeks total per cycle). The primary endpoint was PFS. Secondary endpoints included clinical response rate and characterization of the toxicity profile of this combination therapy.

**Results:**

Nineteen patients were enrolled into the study. The median observed PFS (*n* = 13 evaluable for PFS) was 8.8 months (95% CI 6.8–25.2 months). Best responses achieved were five partial response, nine stable disease, and three disease progression according to RECIST 1.1 guidelines (two non‐evaluable). The most commonly observed toxicities were fatigue, platelet count decrease, creatinine increased, diarrhea, oral mucositis, edema, anemia, rash, hypophosphatemia, dysgeusia, and palmar‐plantar erythrodysesthesia syndrome.

**Conclusion:**

Alternating sunitinib and temsirolimus did not improve the PFS in patients with mRCC.

## 1 INTRODUCTION

Renal cell carcinoma is a highly vascular kidney cancer that is often asymptomatic until late stage, with one third of new cases presenting with metastatic renal cell carcinoma (mRCC). In 2023, the American Cancer Society estimates that approximately 81,800 new cases and 14,890 deaths will be attributed to kidney cancer.^1^ Since 2005, mRCC treatment has seen breakthroughs with the development of inhibitors of vascular endothelial growth factor (VEGF), VEGF receptor (VEGFR), mammalian target of rapamycin (mTOR), and immune checkpoints. First‐line therapy for mRCC with clear cell histology now includes VEFG inhibitors alone or in combination with anti‐PD1, or single agent or dual immune checkpoint inhibition.^2^ Sunitinib is an oral tyrosine kinase inhibitor (TKI) that primarily targets VEGFR, but also inhibits platelet‐derived growth factor receptor (PDGFRs), stem cell factor receptor (Kit), Flt‐3, glial cell line‐derived neurotrophic factor receptor (RET) and colony‐stimulating factor‐1 receptor (CSF‐1R) signaling.^3^ Sunitinib was approved as first‐line therapy for mRCC after increasing progression‐free survival (PFS) from 5 months with standard of care interferon alfa to 11 months.^4^ Temsirolimus binds to the intracellular protein FKBP‐12 and subsequently inhibits mammalian target of rapamycin (mTOR), a mediator of intracellular signaling pathways relating to cell growth and proliferation.^5^ For patients with poor prognosis mRCC, temsirolimus was approved for first‐line therapy with PFS of 4 months and overall survival of 10.9 months.^6^

Due to the success of sunitinib and temsirolimus as single agents, studies were conducted to concomitantly combine the two therapies in an attempt to improve mRCC treatment. However, this combination was poorly tolerated due to observed dose‐limiting toxicities (DLTs) which included mucositis, neutropenia, anemia, thrombocytopenia, rash, and cellulitis.^7^, ^8^ Retrospective studies and a subsequent Phase III trial indicated that temsirolimus treatment following sunitinib was tolerable and provided feasibility for such a sequential therapy regimen in mRCC.^9^, ^10^, ^11^, ^12^ The concept of sequential or alternating therapy is not new,^13^, ^14^, ^15^ and alternating drugs that have different molecular targets and affect independent signaling pathways after each cycle minimize the likelihood of the development of multi‐resistant cancer cells.^16^ Dos Santos et al. treated with alternating sunitinib and everolimus (mTOR inhibitor) every 1, 2, or 3 weeks in a human Caki‐1 RCC xenograft mouse model.^17^ They found all three alternating treatment regimens resulted in longer median time to tumor progression when compared to sunitinib or everolimus monotherapy. The rationale for this clinical trial was to use sunitinib, a TKI, and temsirolimus, an mTOR inhibitor, in an alternating regimen so as to minimize the toxicity observed when combined concomitantly as well as potentially improve the anti‐tumor efficacy compared to monotherapy with either agent.

We therefore hypothesized that alternating sunitinib and temsirolimus therapy would be well tolerated and improve the combined PFS of the agents when used as monotherapy (11 + 4 months). To test this hypothesis we performed a single‐arm, phase II study with alternating 4‐weekly treatment cycles of sunitinib and temsirolimus with a 2‐week rest of no therapy in between each therapy type in patients with mRCC. The primary objective was to determine the PFS of this regimen of alternating therapy in mRCC patients, with secondary objectives including clinical response rate and characterizing the alternating regimen toxicity profile.

## 2 METHODS

### 2.1 Patients

From 2011 to 2014, patients were recruited from the oncology clinics at the Dartmouth Cancer Center and the University of Vermont Cancer Center. Study inclusion criteria were: histologically proven renal cell cancer with measurable metastatic disease according to RECIST 1.1^18^ (both clear cell and non‐clear cell histologies permitted); at least 2 weeks since last immunotherapy, surgery or chemotherapy (6 weeks for nitrosoureas), and recovered from all adverse side effects of prior therapy; Karnofsky performance status^19^ ≥ 60% and life expectancy ≥12 weeks; adequate end organ function with an absolute neutrophil count (ANC) ≥1.5 × 10^9^/L, hemoglobin ≥9 g/dL (subjects may not have had a transfusion within 7 days of screening assessment), platelets ≥100 × 10^9^/L, total bilirubin ≤1.5 × upper limit of normal (ULN, subjects with Gilbert's Syndrome were eligible if their total bilirubin is <3.0 × ULN and direct bilirubin is <3.5), aspartate amino transferase (AST) and alanine amino transferase (ALT) ≤2.5 × ULN, estimated creatinine clearance ≥30 mL/min, and total serum calcium concentration <12.0 mg/dL.

Patients were excluded from the study if they had a concomitant second malignancy except for non‐melanoma skin cancer or non‐invasive cancer, or a prior history of invasive malignancy within 5 years of complete remission. Other exclusion criteria included significant comorbid illness such as uncontrolled diabetes, hypertension or active infection; clinically significant gastrointestinal abnormalities; prolongation of corrected QT interval (QTc) >480 milliseconds; history of any cardiovascular conditions within the past 12 months; prior major surgery or trauma within 28 days prior to first dose of study drug and/or presence of any non‐healing wound, fracture, or ulcer; any serious and/or unstable pre‐existing medical, psychiatric, or other conditions that could interfere with subject's safety, obtaining informed consent, or compliance with the study; evidence of active bleeding or bleeding diathesis; ongoing toxicity from prior anti‐cancer therapy that is >grade 1 and/or that is progressing in severity; previous treatment with either sunitinib or temsirolimus, or known immediate or delayed hypersensitivity reaction to drugs chemically related to sunitinib or temsirolimus; and hemoptysis within 6 weeks of first dose of study drug.

### 2.2 Study design and performance

This was a Phase II, single cohort, open‐label study conducted in compliance with Good Clinical Practice guidelines and the ethical principles of the Declaration of Helsinki. The two clinical study sites were the Dartmouth Cancer Center (Lebanon, NH) and University of Vermont Cancer Center (Burlington, VT) which recruited mRCC patients from these rural state populations. The study protocol and all amendments were reviewed by the Institutional Review Board at each center. The study was registered on clinicaltrials.gov (NCT01517243). Written informed consent was obtained from each patient before any study‐related procedures were performed.

**FIGURE 1:**
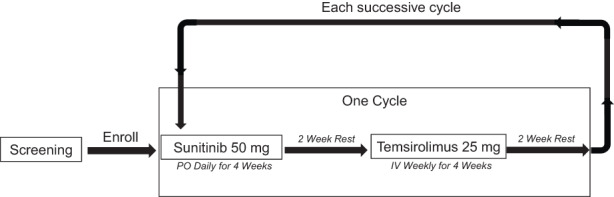
Study design schema.

Patients received sunitinib 50 mg PO daily for 4 weeks followed by a 2‐week rest. Following the rest period, patients received temsirolimus 25 mg IV weekly for 4 weeks followed by a 2‐week rest. This 12‐week dosing regimen of sunitinib followed by temsirolimus constituted one cycle of treatment (Figure 1). Treatment continued until the patient experienced unacceptable toxicity, disease progression, withdrew consent, and/or at physician investigator discretion.

The primary objective of this study was to determine the PFS in mRCC patients treated with this alternating targeted therapy, defined as the time from start of treatment to the first documentation of objective disease progression or to death from any cause, whichever occurred first. Secondary objectives were to determine the clinical response rate and to characterize the toxicity profile of the treatment regimen. Tumor response was evaluated using Response Evaluation Criteria in Solid Tumors, RECIST 1.1 guidelines.^18^

### 2.3 Safety and tolerability assessments

Adverse events (AEs; i.e., toxicity) were carefully monitored during the course of the study by the physician‐investigators. The assessment of safety and tolerability was based on the observed toxicity according to type and grade. Toxicity assessments were made every 6 weeks at the timepoint of beginning the alternate drug treatment within a cycle. Rates of serious grade 3 or worse toxicity were computed as defined by the NCI's Common Terminology Criteria for Adverse Events Version 4.0.^20^ Treatment continued until the patient experienced unacceptable toxicity that precluded further treatment, disease progression, or for up to two cycles following disappearance of all disease, withdrawal of consent, and/or at the discretion of the physician investigator. Patients were allowed to continue therapy after indication of disease progression when the treating physician and study PI were in agreement that the patient was benefiting from therapy.

### 2.4 Tumor burden assessment

Tumor burden was usually assessed using CT imaging. Appropriate imaging evaluations for brain and bone metastases was performed when clinically indicated. RECIST 1.1 criteria were utilized to evaluate the overall response to treatment for patients in this study.^18^ Tumor burden evaluations were performed every 12 weeks (+/−7 days) while on study treatment.

### 2.5 Statistics

In this single‐arm open‐label study, the primary endpoint of PFS was determined using Kaplan–Meier method for survival.^21^ We used a nonparametric sample size calculation with a one‐sided test, an alpha of 0.05 and a power of 90% for a total sample size of 37 evaluable patients needed to be able to reject the null hypothesis of no improvement compared to an expected 15 month PFS for the combination based on additive benefit.

Due to slower than expected accrual, a prospective, but initially unplanned interim analysis for futility of achieving the projected PFS increase from 15 to 30 months by continuing to enroll the additional 24 patients evaluable for PFS was performed after 13 evaluable patients were enrolled using the Weibull distribution^22^ followed by a Kaplan–Meier analysis.

## 3 RESULTS

### 3.1 Patients and baseline demographic characteristics

Nineteen patients were enrolled in the study and all of them received study treatment. The study was terminated early due to the changing landscape of therapies and the lack of a clear signal of benefit in the first 13 patients evaluable for PFS. Seventeen patients received at least one full cycle of study treatment. The patients on this trial had similar characteristics to the national averages in the United States.^1^ Study subjects were predominantly male (twice as common in men compared to women), White, non‐Hispanic, with a median age of 64 (64 is the average age of people when they are diagnosed).^1^ In terms of tumor histology, 16 out of 19 patients (84.2%) had clear cell RCC, which is comparable to the reported 75%–80% of RCC cases having clear cell histology.^23^ Detailed patient demographics of patients enrolled in the study are shown in Table 1. Detailed information regarding the metastatic sites of the patients is found in Table 2.

**TABLE 1:** Detailed demographics for the enrolled patient population (*n* = 19).

Characteristic	Category	Enrolled patients
Sex, *n* (%)	Male	16 (84.2)
	Female	3 (15.8)
Age, years	Median	64
	Range	48–83
Age, *n* (%)	<65 years	10 (52.6)
	≥65 years	9 (47.4)
Race, *n* (%)	White	19 (100)
Ethnicity, *n* (%)	Other (non‐Hispanic/Latino)	19 (100)
Karnofsky performance score (%)	Mean (SD)	84.2 (10.2)
	Median	80
	Range	70–100
Tumor histology, *n* (%)	Clear Cell	16 (84.2)
	Papillary	1 (5.3)
	Unclassified	2 (10.5)

**TABLE 2:** Metastatic sites of the enrolled patient population (*n* = 19).

Metastatic sites	Number of patients
Lung +/− mesenteric nodes +/− Bone	5
Lung and mediastinal nodes +/− Bone	5
Lung and brain + Liver or thyroid	2
Lung only	2
Lung and liver +/− Pancreas	2
Mediastinal nodes and bone	1
Mesenteric nodes and chest wall	1
Pancreas only	1

### 3.2 Study treatment and clinical response

**FIGURE 2:**
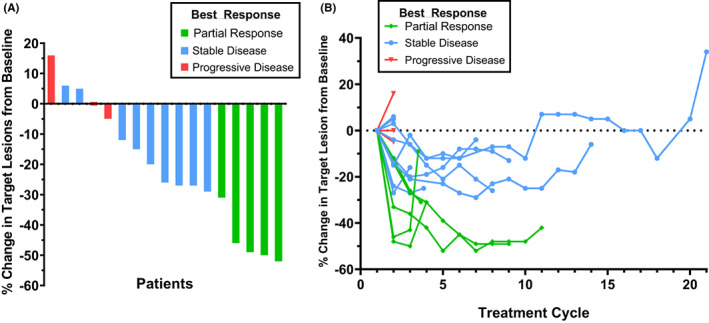
(A) Patients best response from baseline as a waterfall plot (*n* = 17). The two patients with little or no decrease in % change of tumor size from baseline and judged to have progressive disease had the appearance of new lesions or unequivocal increase in non‐target lesions. (B) Timeline of patient tumor response from baseline as a spider plot (*n* = 17)

Best responses achieved by patients enrolled in the study and the corresponding % change in target lesions from baseline are illustrated as a waterfall plot in Figure 2A. Change in tumor burden over the course of study treatment compared to baseline is visually presented for each patient in the spider plot shown in Figure 2B. Seventeen patients were evaluable for tumor response, the best responses achieved were five partial responses (26.3%), nine with initial stable disease (47.4%), and three disease progression (15.8%). Two patients (10.5%) were not evaluable for tumor response because they came off study before completing a full cycle consisting of both drug treatments. A summary of the study outcomes for the 19 enrolled patients is found in Table 3.

**TABLE 3:** Duration on study treatment and clinical response (*n* = 19 enrolled).

Study outcomes		All Patients
Number of patients enrolled		19
# Cycles completed	Median (Range)	2 (0–20)
# Months on study	Median (Range)	7.7 (0.8–59.4)
Best response achieved, *n* (%)	PR	5 (26.3)
	SD	9 (47.4)
	PD	3 (15.8)
	NE	2 (10.5)
PFS (months)	Median (95% CI)	8.8 (6.8–25.2)

Abbreviations: NE, not evaluable; PD, progression of disease; PFS, progression‐free survival; PR, partial response; SD, stable disease.

Twelve of the 19 enrolled patients (63.2%) ultimately came off study due to disease progression and one patient died while on study. Of the 12 patients with disease progression, 4 were due to a >20% increase in sum of the long diameters of target lesions and 8 were due to the appearance of new or unequivocal progression of non‐target lesions. Six patients discontinued treatment due to AEs. The serious adverse events (SAEs) or AEs that caused patients to come off study included sunitinib grade 3/4 toxicity (2), grade 3 pneumonitis due to temsirolimus (1), grade 4 stroke possibly related to sunitinib (1), being off sunitinib for an extended period due to dental pain needing multiple teeth extractions (1), and one grade 5 toxicity‐death (1). One patient voluntarily withdrew from the study after completing 8 cycles of study treatment and achieving a partial response.

**FIGURE 3:**
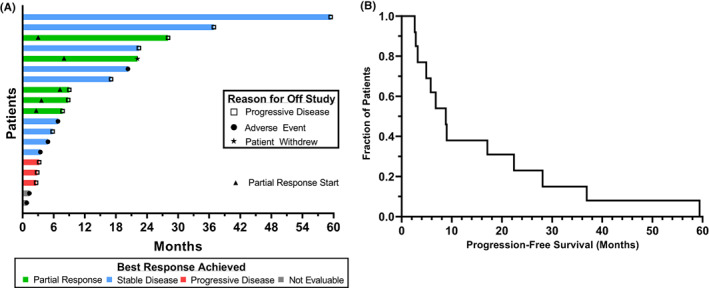
(A) Time on study treatment, reason for off study, and partial response start as a Swimmer plot (*n* = 19). (B) Kaplan–Meier plot for time to progression‐free survival (*n* = 13)

The median treatment duration was 7.7 months (range 0.8–59.4). Patients received a median of 2 cycles (range 0–20) of study treatment. Treatment duration, changes in clinical response and reasons for treatment discontinuation are presented in Figure 3A. For the 13 evaluable patients in this study, the median PFS was 8.8 months (range 2.6–59.4). The PFS data are illustrated in the Kaplan–Meier plot shown in Figure 3B.

Only 19 patients out of the planned 37 patients were accrued to this clinical trial. The study was stopped before reaching the enrollment goal due primarily to the emergence of newer therapies for mRCC which slowed the rate and ability to accrue additional patients into the study. Furthermore, we undertook an unplanned interim analysis and imputed the PFS for the proposed remaining study cohort population *n* = 24 using a Weibull distribution,^22^ with parameters estimated based on the enrolled evaluable 13 patients with the PFS data. We then estimated the median survival time of the combined data (*n* = 37) using Kaplan–Meier method. We repeated the same procedure for 1000 times and found the average median survival time to be 17 months which is slightly better than historical literature‐based control median PFS of 15 months. Interestingly, the estimated median survival time in 533 of the 1000 imputations was <15 months and most notably none of the PFS estimates in these simulations were over 30 months which was the estimated PFS that was used to adequately power this study. We therefore concluded that further enrollment into the study would be futile. All analysis was done in R.^24^

### 3.3 Safety, toxicity, and adverse events

SAEs occurred in four of the nineteen enrolled patients (21.1%). Events of grade ≥3 were experienced by 73.7% of patients on the study. Three of the SAEs that occurred were a cerebral ischemia event (stroke) in one patient that also caused right‐sided muscle weakness and dysarthria (all grade 4) which were all considered possibly related to sunitinib and resulted in this patient coming off the study. Another patient experienced grade 2 atrial fibrillation considered possibly related to sunitinib which resolved after a day and the patient remained on study. One death occurred while a patient was on cycle 2 of study treatment which was not considered related to either drug. This was a suspected myocardial infarction but there was no autopsy performed that would have confirmed this. One patient experienced grade 3 urosepsis requiring hospitalization for 2 days. The patient had a history of urinary tract infections and the urosepsis was not related to either drug. Two years later, the same patient experienced grade 3 pneumonia with sepsis requiring hospitalization for 6 days, considered likely related to temsirolimus. Following each of these SAEs, this patient recovered and was continued on treatment.

Treatment‐emergent AEs were any AEs considered possibly, probably or definitely related to either drug. Treatment‐emergent AEs of some grade occurred in all patients. AEs were consistent with those expected for sunitinib or temsirolimus alone. Treatment‐emergent AEs experienced by >10% of patients are detailed in Table 4. The most common treatment‐emergent AEs were (*n* of total observed): fatigue (17), platelet count decreased (12), creatinine increased (12), diarrhea (10), oral mucositis (10), limb edema (9), anemia (8), rash (8), hypophosphatemia (7), dysgeusia (7), and palmar‐plantar erythrodysesthesia syndrome (7). A greater total number of AEs were related to sunitinib (117) than temsirolimus (65). There were multiple AEs judged potentially related to both drugs (55).

**TABLE 4:** Detailed table of treatment‐related AEs which were observed in >10% patients, their grade, and relationship to drug (S = sunitinib; T = temsirolimus; B = both).

Treatment‐emergent adverse events >10% of patients		CTCAE v4.0 Grade											
		Grade 1			Grade 2			Grade 3			Grade 4		
Total patients with AEs, *n* (%)		18 (94.7%)			18 (94.7%)			13 (68.4%)			5 (26.3%)		
Treatment emergent AEs > 10% of patients (i.e., more than one patient)		S	T	B	S	T	B	S	T	B	S	T	B
Blood and lymphatic system Disorders	Anemia	1	‐	6	‐	‐	‐	‐	‐	1	‐	‐	‐
	Thrombocytopenia	‐	2	‐	‐	‐	‐	‐	‐	‐	1	‐	‐
Gastrointestinal disorders	Diarrhea	5	‐	1	2	‐	1	1	‐	‐	‐	‐	‐
	Dyspepsia	2	‐	‐	1	‐	‐	‐	‐	‐	‐	‐	‐
	Mucositis oral	3	3	1	‐	1	2	‐	‐	‐	‐	‐	‐
	Nausea	4	‐	1	‐	‐	‐	‐	‐	‐	‐	‐	‐
	Vomiting	1	‐	‐	‐	‐	1	1	‐	‐	‐	‐	‐
General disorders and administration site conditions	Edema limbs	2	1	1	1	3	1	‐	‐	‐	‐	‐	‐
	Facial pain	‐	‐	‐	2	‐	‐	‐	‐	‐	‐	‐	‐
	Fatigue	2	‐	9	1	1	2	2	‐	‐	‐	‐	‐
Immune system disorders	Allergic reaction (Rash)	3	2	2	1	‐	‐	‐	‐	‐	‐	‐	‐
investigations	Alanine aminotransferase Increased	1	3	‐	‐	‐	‐	‐	‐	‐	‐	‐	‐
	Alkaline phosphatase increased	1	1	1	‐	‐	‐	‐	‐	‐	‐	‐	‐
	Aspartate aminotransferase increased	1	1	‐	1	‐	‐	‐	‐	‐	‐	‐	‐
	Blood bilirubin increased	2	‐	‐	‐	‐	‐	‐	‐	‐	‐	‐	‐
	Creatinine increased	1	4	1	2	2	2	‐	‐	‐	‐	‐	‐
	Lipase increased	‐	‐	‐	‐	‐	‐	2	‐	‐	‐	‐	‐
	Neutrophil count decreased	‐	‐	‐	‐	‐	‐	‐	2	‐	‐	‐	‐
	Platelet count decreased	3	5	3	‐	‐	‐	1	‐	‐	‐	‐	‐
	Serum amylase increased	2	1	‐	‐	‐	‐	1	‐	‐	‐	‐	‐
	Weight loss	2	‐	‐	‐	‐	1	‐	‐	‐	‐	‐	‐
	Anorexia	1	‐	1	1	‐	‐	‐	‐	‐	‐	‐	‐
Metabolism and nutrition disorders	Hyperglycemia	1	‐	1	‐	‐	‐	‐	1	‐	‐	‐	‐
	Hypertriglyceridemia	‐	1	‐	‐	‐	‐	‐	‐	‐	‐	1	‐
	Hypoalbuminemia	1	‐	‐	‐	‐	1	‐	‐	‐	‐	‐	‐
	Hypokalemia	‐	1	‐	‐	‐	‐	‐	1	‐	‐	‐	‐
	Hyponatremia	1	1	1	‐	‐	‐	1	‐	‐	‐	‐	‐
	Hypophosphatemia	2	‐	‐	2	‐	‐	1	2	‐	‐	‐	‐
Musculoskeletal and connective tissue disorders	Myalgia	2	‐	‐	‐	‐	‐	‐	‐	‐	‐	‐	‐
Nervous system disorders	Dysesthesia	2	‐	‐	‐	‐	‐	‐	‐	‐	‐	‐	‐
	Dysgeusia	6	‐	1	‐	‐	‐	‐	‐	‐	‐	‐	‐
	Headache	1	1	1	‐	‐	‐	‐	‐	‐	‐	‐	‐
	Sinus pain	‐	2	‐	‐	‐	‐	‐	‐	‐	‐	‐	‐
	Chronic kidney disease	‐	‐	1	‐	‐	1	‐	‐	‐	‐	‐	‐
Renal and urinary disorders	Proteinuria	1	‐	1	1	‐	‐	‐	‐	‐	‐	‐	‐
	Cough	‐	3	‐	‐	‐	‐	‐	‐	‐	‐	‐	‐
Respiratory, thoracic and mediastinal disorders	Dyspnea	‐	2	‐	‐	‐	‐	‐	‐	‐	‐	‐	‐
	Epistaxis	2	‐	‐	‐	‐	‐	‐	‐	‐	‐	‐	‐
	Pleural effusion	2	‐	‐	‐	‐	‐	‐	‐	‐	‐	‐	‐
Skin and subcutaneous tissue disorders	Palmar‐Plantar Erythrodysesthesia syndrome	3	‐	1	3	‐	‐	‐	‐	‐	‐	‐	‐
	Pruritus	‐	2	‐	‐	‐	‐	‐	‐	‐	‐	‐	‐
	Rash acneiform	‐	1	1	‐	‐	‐	‐	‐	‐	‐	‐	‐
Vascular disorders	Hypertension	1	1	‐	2	‐	‐	2	‐	‐	‐	‐	‐

## 4 DISCUSSION

To our knowledge, this is the first study which evaluated whether alternating sunitinib and temsirolimus therapies could potentially increase the PFS in patients with mRCC and be reasonably well tolerated. The choice of sunitinib and temsirolimus here is important because there has not been any data published on this specific alternating regimen. In vitro studies of temsirolimus in renal cell carcinoma cell lines showed reduction of hypoxia‐inducible factors HIF‐1 and HIF‐2 alpha, and the vascular endothelial growth factor (VEGF). By reducing VEGF, temsirolimus reduces target competition for sunitinib which binds the VEGF receptor and could potentially therefore increase efficacy of combining sunitinib with temsirolimus in this treatment regimen. VEGF and mTOR converge at HIF and VEGFR inhibitors are more effective in clear cell RCC (75%–80% of RCC are clear cell^23^) where Von Hippel Lindau (VHL) genes are mutated or deleted.^25^ VHL genes provide instructions for making a protein that functions as part of a protein complex called the VCB‐CUL2 complex. This complex targets other proteins to be degraded by the cell when they are no longer needed and is a normal process that helps maintain normal cell function.

Although our study did not evaluate immunological parameters, the underlying rationale for potential synergistic immunological effects of sunitinib and temsirolimus has been subsequently supported by a study comparing the immunological impact of sunitinib, everolimus, and temsirolimus which indicated that temsirolimus was expected to offer the best outcome with immunomodulators in RCC.^26^ Other mTOR inhibitors such as everolimus demonstrated an increase in dysfunctional CD8+ T cells, while temsirolimus decreased PD‐1 expression of CD8+ T cells and early‐stage MDSC and increased interferon‐γ and TNF‐α production. Sunitinib decreased early‐stage MDSCs while increasing the number of natural killer cells without impacting CD4+ or CD8+ cells.^26^ Recognizing the role of mTOR and VEGF on immunologic targets provides further rational development of this targeted combination and immune therapies beyond those already approved for mRCC.

Since the onset of this trial, other studies have been conducted that tested the efficacy and safety of alternating TKIs and mTOR inhibitors with similar results. Two phase II trials have studied alternating sunitinib and everolimus, an mTOR inhibitor, as first‐line therapy for metastatic RCC. A single‐arm phase II trial in advanced RCC (EVERSUN) investigated a 12‐week alternating regimen cycle consisting of sunitinib 4 weeks on, 2 weeks off, followed by everolimus 5 weeks on, 1 week off schedule.^27^ Patients treated with this regimen had an observed median PFS of 8 months. In a similar randomized phase II study (SUNRISES), the experimental alternating regimen cycle consisted of 12 weeks sunitinib (4 weeks on, 2 weeks off) followed by 12 weeks of everolimus (11 weeks on, 1 week off).^28^ The experimental arm had a median PFS of 10 months compared to 25.07 months for the control arm of sunitinib until progression followed by everolimus. In the ROPETAR study, an alternating treatment regimen of 8 weeks of pazopanib, a TKI, followed by 8 weeks of everolimus failed to prolong PFS.^29^ Patients treated on this alternating regimen had a median PFS of 7.4 months compared to 9.4 months for pazopanib therapy. The authors of this study also found no clinical benefit in terms of toxic effects or quality of life. The observed median PFS of 8.8 months for the alternating regimen of sunitinib and temsirolimus tested in our clinical trial is similar to these three other studies.

There have also been studies of alternating TKIs with bevacizumab, a monoclonal antibody that targets VEGF. A phase I/II trial of patients with advanced clear cell RCC tested the standard 4 weeks on, 2 weeks off sunitinib regimen with the addition of bevacizumab on Day 29 of each sunitinib cycle.^30^ The rationale behind this treatment regimen was that the 2‐week rest period of standard sunitinib therapy could result in rebound angiogenesis and tumor progression. As was the case with our study, this study closed prematurely due to poor accrual but in the 25 patients recruited, a median PFS was reported to be 16.5 months. The authors of this study noted an improved efficacy over sunitinib monotherapy but this came at the expense of increased, though manageable, toxicity. A phase I/II trial in treatment‐naïve metastatic clear cell RCC patients studied a 10‐week cycle consisting of pazopanib on Days 1–28 with bevacizumab on Days 36 and 50.^31^ The observed median PFS was 20.9 months for the 25 patients enrolled, suggesting this novel regimen could be tested in a phase II trial. Treatments for mRCC have moved onto the era of immune checkpoint inhibition with anti‐PD‐1/PD‐L1 or anti CTLA‐4 blocking antibodies demonstrating generally very effective results. A Phase III trial (CheckMate 214) of nivolumab plus ipilimumab found overall survival and objective response rates were significantly higher with nivolumab plus ipilimumab than with sunitinib among intermediate and poor risk patients with previously untreated advanced RCC.^32^ Median PFS was 11.6 months on nivolumab plus ipilimumab versus 8.4 months on sunitinib. In April 2018, the FDA approved nivolumab plus ipilimumab in combination for the treatment of intermediate or poor risk, previously untreated advanced RCC.

A Phase III trial in untreated advanced RCC (KEYNOTE‐426) revealed pembrolizumab (anti‐PD‐1) plus axitinib (anti‐VEGFR) with PFS of 15.1 months and ORR 59.3% was more effective than sunitinib with PFS of 11.1 months and ORR 35.7%. This benefit was seen across all risk groups regardless of PD‐L1 expression.^33^ In April 2019, the FDA approved pembrolizumab plus axitinib as a first‐line treatment for patients with advanced RCC. A parallel‐cohort, dose‐escalation, phase I CheckMate 016 study evaluated the efficacy and safety of nivolumab in combination with either sunitinib or pazopanib or ipilimumab.^34^ CheckMate 016 concluded the addition of standard doses of sunitinib or pazopanib to nivolumab resulted in a high incidence of high‐grade toxicities limiting future development of either combination regimen.

A phase III trial in untreated advanced RCC (CheckMate 9ER) demonstrated nivolumab (anti‐PD1) plus cabozantinib (anti‐VEGFR) was safe and effective with a median PFS of 16.6 months and 85.7% overall survival (OS) at 12 months compared to PFS of 8.3 months and OS at 12 months of 75.6% for sunitinib.^35^ In January 2021, the FDA approved the combination of nivolumab plus cabozantinib as another regimen for first‐line treatment for patients with advanced RCC. Later that year, in August 2021, the FDA approved the combination of lenvatinib (anti‐VEGFR) and pembrolizumab (anti‐PD1) based on the results of another phase III trial in advanced RCC (CLEAR).^36^ The CLEAR trial found a median PFS of 23.9 months for lenvatinib plus pembrolizumab compared to 14.7 months for lenvatinib plus everolimus and 9.2 months for sunitinib monotherapy. Compared to sunitinib monotherapy, overall survival was longer with lenvatinib plus pembrolizumab but not with lenvatinib plus everolimus. The KEYNOTE‐426, CheckMate 214, CheckMate 9ER, and CLEAR trials all used the same comparator of sunitinib, which had been the standard of care at the time of these trials. Head‐to‐head trials of these regimens have not been conducted.

The results of these landmark trials have confirmed that immune checkpoint inhibitor combined with VEGFR‐TKI therapy is the standard of care in treating patients with advanced mRCC. National Comprehensive Cancer Network (NCCN) clinical practice guidelines for kidney cancer state preferred regimens for first‐line treatment in patients with clear cell RCC as axitinib with pembrolizumab, cabozantinib with nivolumab, and lenvatinib with pembrolizumab for all risk groups.^2^ For patients with poor or intermediate risk, preferred regimens include ipilimumab with nivolumab and cabozantinib monotherapy in addition to the three regimens outlined for all risk groups. Other recommended regimens include axitinib with avelumab, pazopanib, and sunitinib. Because response rates in non‐clear cell RCC (nccRCC) are significantly lower, the preferred strategy for patients with nccRCC is enrollment in clinical trials. However, preferred approved regimens include cabozantinib and sunitinib.

Our study was terminated prior to completion of enrollment due to a slow rate of accrual impacted by the emergence of the aforementioned therapies as well as the previously described interim analysis that showed no improvement in PFS over historical data even if the study enrolled the total projected population of *n* = 37 patients. The lack of racial diversity in this study population is another limitation but it reflects the general population of the rural states of Vermont and New Hampshire from which the study patients were enrolled. Populations living in rural areas have higher average cancer death rates compared to populations in urban areas and therefore rural communities represent an underserved population according to the National Cancer Institute.^37^

In conclusion, although the alternating schedule of sunitinib and temsirolimus can be given to patients relatively safely, the regimen did not improve the median PFS in patients with mRCC. The standard of care for mRCC has changed considerably since we first started enrolling patients into the study in 2011 with improvements in PFS and overall survival. With the current widespread use of additional therapeutic options particularly immune checkpoint inhibitors and the futility analysis performed on our study data for prolonging PFS, the alternating regimen of sunitinib–temsirolimus is not recommended for further study in larger clinical trials. This recommendation is based not only on the results of our study and but other clinical trials, where the treatment approach of alternating TKIs and mTOR inhibitors has not shown an improvement in clinical outcomes for patients with mRCC and therefore does not warrant additional investigation. Nevertheless, the observations of two patients with PFS of 36.9 and 59.4 months in this study support the evaluation of exceptional responders to better appreciate the mechanistic heterogeneity of therapeutic pathways.^38^, ^39^

## AUTHOR CONTRIBUTIONS

**Dylan B. Ness:** Data curation (lead); formal analysis (equal); visualization (equal); writing – original draft (lead); writing – review and editing (lead). **Darcy B. Pooler:** Data curation (equal); formal analysis (equal); visualization (equal); writing – original draft (equal); writing – review and editing (equal). **Steven Ades:** Investigation (equal); project administration (equal); supervision (equal); writing – review and editing (equal). **Brian J. Highhouse:** Investigation (equal); project administration (equal); writing – review and editing (equal). **Bridget M. Labrie:** Data curation (equal); project administration (equal); supervision (equal); writing – review and editing (equal). **Jie Zhou:** Formal analysis (equal); software (equal); writing – review and editing (equal). **Jiang Gui:** Formal analysis (equal); methodology (equal); software (equal); writing – review and editing (equal). **Lionel D. Lewis:** Conceptualization (equal); investigation (equal); methodology (equal); project administration (equal); supervision (equal); writing – original draft (equal); writing – review and editing (equal). **Marc S. Ernstoff:** Conceptualization (lead); funding acquisition (equal); investigation (equal); methodology (equal); project administration (equal); supervision (equal); writing – original draft (equal); writing – review and editing (equal).

## FUNDING INFORMATION

Dartmouth Cancer Center Support Grant P30CA023108.

## CONFLICT OF INTEREST STATEMENT

LDL is a consultant to G1Therapeutics and 7 Hills Pharma LLC and receives funding via awards to Dartmouth‐Health for clinical trials from Bristol Myers Squibb, AbbVie, Astra Zeneca, Bayer Pharmaceuticals and Curis Inc. The other authors have no conflicts to declare.

## PATIENT CONSENT

Written informed consent was obtained from each patient before any study‐related procedures were performed.

## CLINICAL TRIAL REGISTRATION

The study was registered on clinicaltrials.gov (NCT01517243).

## Data Availability

Requests for de‐identified data may be directed to Dylan B. Ness (dylan.b.ness@hitchcock.org).

## References

[1] American Cancer Society . Key Statistics About Kidney Cancer. Accessed March 7, 2023, https://www.cancer.org/cancer/kidney‐cancer/about/key‐statistics.html#references

[2] Motzer RJ , Jonasch E , Agarwal N , et al. Kidney cancer, version 3.2022, NCCN clinical practice guidelines in oncology. J Natl Compr Canc Netw. 2022;20(1):71‐90. doi:10.6004/jnccn.2022.0001 34991070 PMC10191161

[3] SUTENT (sunitinib malate) capsules, for oral use (FDA) (2017).

[4] Motzer RJ , Hutson TE , Tomczak P , et al. Sunitinib versus interferon alfa in metastatic renal‐cell carcinoma. N Engl J Med. 2007;356(2):115‐124. doi:10.1056/NEJMoa065044 17215529

[5] Fingar DC , Richardson CJ , Tee AR , Cheatham L , Tsou C , Blenis J . mTOR controls cell cycle progression through its cell growth effectors S6K1 and 4E‐BP1/eukaryotic translation initiation factor 4E. Mol Cell Biol. 2004;24(1):200‐216. doi:10.1128/mcb.24.1.200-216.2004 14673156 PMC303352

[6] Hudes G , Carducci M , Tomczak P , et al. Temsirolimus, interferon alfa, or both for advanced renal‐cell carcinoma. N Engl J Med. 2007;356(22):2271‐2281. doi:10.1056/NEJMoa066838 17538086

[7] Campbell MT , Millikan RE , Altinmakas E , et al. Phase I trial of sunitinib and temsirolimus in metastatic renal cell carcinoma. Clin Genitourin Cancer. 2015;13(3):218‐224. doi:10.1016/j.clgc.2014.10.004 25465491 PMC4991027

[8] Patel PH , Senico PL , Curiel RE , Motzer RJ . Phase I study combining treatment with temsirolimus and sunitinib malate in patients with advanced renal cell carcinoma. Clin Genitourin Cancer. 2009;7(1):24‐27. doi:10.3816/CGC.2009.n.004 19213664 PMC3740755

[9] Gerullis H , Bergmann L , Maute L , et al. Feasibility of sequential use of sunitinib and temsirolimus in advanced renal cell carcinoma. Med Oncol. 2010;27(2):373‐378. doi:10.1007/s12032-009-9220-1 19399651

[10] Grundbichler M , Mlineritsch B , Ressler S , et al. Efficacy of temsirolimus after previous treatment with sunitinib, sorafenib or everolimus in advanced renal cell cancer. Oncology. 2011;80(1–2):34‐41. doi:10.1159/000328086 21606662

[11] Iacovelli R , Carteni G , Milella M , et al. Clinical outcomes in patients with metastatic renal cell carcinoma receiving everolimus or temsirolimus after sunitinib. Can Urol Assoc J. 2014;8(3–4):E121‐E125. doi:10.5489/cuaj.1604 24678349 PMC3956829

[12] Hutson TE , Escudier B , Esteban E , et al. Randomized phase III trial of temsirolimus versus sorafenib as second‐line therapy after sunitinib in patients with metastatic renal cell carcinoma. J Clin Oncol. 2014;32(8):760‐767. doi:10.1200/JCO.2013.50.3961 24297950 PMC5569683

[13] Eagan RT , Frytak S , Richardson RL , et al. A randomized comparative trial of sequential versus alternating cyclophosphamide, doxorubicin, and cisplatin and mitomycin, lomustine, and methotrexate in metastatic non‐small‐cell lung cancer. J Clin Oncol. 1988;6(1):5‐8. doi:10.1200/JCO.1988.6.1.5 2826714

[14] Perol M , Lena H , Thomas P , et al. Phase II randomized multicenter study evaluating a treatment regimen alternating docetaxel and cisplatin‐vinorelbine with a cisplatin‐vinorelbine control group in patients with stage IV non‐small‐cell lung cancer: GFPC 97.01 study. Ann Oncol. 2002;13(5):742‐747. doi:10.1093/annonc/mdf128 12075743

[15] Bonadonna G , Zambetti M , Valagussa P . Sequential or alternating doxorubicin and CMF regimens in breast cancer with more than three positive nodes. Ten‐year results. JAMA. 1995;273(7):542‐547. 7837388

[16] Goldie JH , Coldman AJ . A mathematic model for relating the drug sensitivity of tumors to their spontaneous mutation rate. Cancer Treat Rep. 1979;63(11–12):1727‐1733. 526911

[17] Santos CD , Tijeras‐Raballand A , Serova M , et al. Effects of preset sequential administrations of sunitinib and everolimus on tumour differentiation in Caki‐1 renal cell carcinoma. Br J Cancer. 2015;112(1):86‐94. doi:10.1038/bjc.2014.578 25422908 PMC4453618

[18] Eisenhauer EA , Therasse P , Bogaerts J , et al. New response evaluation criteria in solid tumours: revised RECIST guideline (version 1.1). Eur J Cancer. 2009;45(2):228‐247. doi:10.1016/j.ejca.2008.10.026 19097774

[19] Karnofsky DA , Burchenal JH . The clinical evaluation of chemotherapeutic agents in cancer. In: MacLeod CM , ed. Evaluation of Chemotherapeutic Agents. Columbia University Press; 1949:191‐205.

[20] Common Terminology Criteria for Adverse Events (CTCAE) . Version 4.0 (US Department of Health and Human Services). 2009.

[21] Ornstein MC , Pal SK , Wood LS , et al. Individualised axitinib regimen for patients with metastatic renal cell carcinoma after treatment with checkpoint inhibitors: a multicentre, single‐arm, phase 2 study. Lancet Oncol. 2019;20(10):1386‐1394. doi:10.1016/s1470-2045(19)30513-3 31427205

[22] Johnson NL , Kotz S , Balakrishnan N . Continuous univariate distributions. Probability and Mathematical Statistics: Applied Probability and Statistics. 2nd ed. Wiley Series John Wiley & Sons, Inc; 1994.

[23] Cairns P . Renal cell carcinoma. Cancer Biomark. 2010;9(1–6):461‐473. doi:10.3233/cbm-2011-0176 22112490 PMC3308682

[24] R: A Language and Environment for Statistical Computing . R Foundation for Statistical Computing. 2022 http://www.R‐project.org

[25] Rini BI . Metastatic renal cell carcinoma: many treatment options, one patient. J Clin Oncol. 2009;27(19):3225‐3234. doi:10.1200/JCO.2008.19.9836 19470934

[26] Kobayashi Y , Yamada D , Kawai T , et al. Different immunological effects of the molecular targeted agents sunitinib, everolimus and temsirolimus in patients with renal cell carcinoma. Int J Oncol. 2020;56(4):999‐1013. doi:10.3892/ijo.2020.4975 32319571

[27] Davis ID , Long A , Yip S , et al. EVERSUN: a phase 2 trial of alternating sunitinib and everolimus as first‐line therapy for advanced renal cell carcinoma. Ann Oncol. 2015;26(6):1118‐1123. doi:10.1093/annonc/mdv078 25701452

[28] Rodriguez‐Vida A , Bamias A , Esteban E , et al. Randomised phase II study comparing alternating cycles of sunitinib and everolimus vs standard sequential administration in first‐line metastatic renal carcinoma (SUNRISES study). BJU Int. 2020;126(5):559‐567. doi:10.1111/bju.15165 32654362

[29] Cirkel GA , Hamberg P , Sleijfer S , et al. Alternating treatment with pazopanib and Everolimus vs continuous pazopanib to delay disease progression in patients with metastatic clear cell renal cell cancer: the ROPETAR randomized clinical trial. JAMA Oncol. 2017;3(4):501‐508. doi:10.1001/jamaoncol.2016.5202 27918762

[30] Bazarbashi S , Alzahrani A , Aljubran A , et al. Combining sunitinib and bevacizumab for the Management of Advanced Renal Cell Carcinoma: a phase I/II trial. Oncologist. 2023. doi:10.1093/oncolo/oyac261 PMC10166178 36648325

[31] George S , Herbst L , Sikorski M , et al. A phase I/II trial of pazopanib alternating with bevacizumab in treatment‐naïve metastatic clear cell renal cell carcinoma (CCRCC) patients: phase I results. J Clin Oncol. 2019;37(7_suppl):561. doi:10.1200/JCO.2019.37.7_suppl.561

[32] Motzer RJ , Tannir NM , McDermott DF , et al. Nivolumab plus ipilimumab versus sunitinib in advanced renal‐cell carcinoma. N Engl J Med. 2018;378(14):1277‐1290. doi:10.1056/NEJMoa1712126 29562145 PMC5972549

[33] Rini BI , Plimack ER , Stus V , et al. Pembrolizumab plus Axitinib versus sunitinib for advanced renal‐cell carcinoma. N Engl J Med. 2019;380(12):1116‐1127. doi:10.1056/NEJMoa1816714 30779529

[34] Amin A , Plimack ER , Ernstoff MS , et al. Safety and efficacy of nivolumab in combination with sunitinib or pazopanib in advanced or metastatic renal cell carcinoma: the CheckMate 016 study. J ImmunoTher Cancer. 2018;6(1):109. doi:10.1186/s40425-018-0420-0 30348216 PMC6196426

[35] Choueiri TK , Powles T , Burotto M , et al. Nivolumab plus Cabozantinib versus sunitinib for advanced renal‐cell carcinoma. N Engl J Med. 2021;384(9):829‐841. doi:10.1056/NEJMoa2026982 33657295 PMC8436591

[36] Motzer R , Alekseev B , Rha S‐Y , et al. Lenvatinib plus pembrolizumab or Everolimus for advanced renal cell carcinoma. N Engl J Med. 2021;384(14):1289‐1300. doi:10.1056/NEJMoa2035716 33616314

[37] Kennedy AE , Vanderpool RC , Croyle RT , Srinivasan S . An overview of the National Cancer Institute's initiatives to accelerate rural cancer control research. Cancer Epidemiol Biomarkers Prev. 2018;27(11):1240‐1244. doi:10.1158/1055-9965.Epi-18-0934 30385495 PMC13577081

[38] Elzein A , Iyer G , Solit DB . Lessons from the study of exceptional responders. Cancer Cell. 2021;39(1):11‐13. doi:10.1016/j.ccell.2020.11.008 33434508

[39] Wheeler DA , Takebe N , Hinoue T , et al. Molecular features of cancers exhibiting exceptional responses to treatment. Cancer Cell. 2021;39(1):38‐53.e7. doi:10.1016/j.ccell.2020.10.015 33217343 PMC8478080

